# Drug-Induced Liver Injury Secondary to Endocrine Therapy With Aromatase Inhibitors: A Case Report

**DOI:** 10.7759/cureus.80795

**Published:** 2025-03-18

**Authors:** Nicholas J Smith, Sariah Watchalotone, Sonia Sandhu

**Affiliations:** 1 Oncology, Midwestern University Arizona College of Osteopathic Medicine, Glendale, USA; 2 Oncology, Ironwood Cancer and Research Centers, Glendale, USA

**Keywords:** adjuvant endocrine therapy, anastrozole, aromatase inhibitors, drug-induced liver injury (dili), hormone receptor-positive breast cancer

## Abstract

Aromatase inhibitors (AIs) are routinely used in treating estrogen receptor (ER)-positive breast cancer in postmenopausal women and are effective in reducing the recurrence of metastatic hormone-sensitive breast cancer. We present the case of a 76-year-old female with a past medical history of hypothyroidism and dyslipidemia who presented for the treatment of ER-positive pleomorphic lobular carcinoma in situ (LCIS). After initiating anastrozole, she developed weakness, loss of appetite, darkening of urine, and jaundice. Upon admission to the hospital, results from the patient’s initial workup revealed abnormal liver function tests. Anastrozole was identified as the causative agent for hepatic injury and was discontinued. The patient’s liver enzymes normalized two months off of the medication, and she decided to proceed with routine observation rather than initiating new endocrine therapy. This case emphasizes the importance of recognizing drug-induced liver injury (DILI) as a rare and serious complication of anastrozole use and highlights the need for consideration of possible risks with endocrine therapies.

## Introduction

Breast cancer is the most commonly diagnosed cancer among women in the United States and is the second most likely cause of cancer death in women [1]. Aromatase inhibitors (AIs) are a class of drugs frequently used in adjuvant endocrine therapy for the treatment of ER-positive breast cancer in postmenopausal women [2]. AIs, such as anastrozole, letrozole, and exemestane, inhibit aromatase, an enzyme necessary for estrogen synthesis, thereby decreasing estrogen availability to estrogen-dependent breast cancer cells [2]. Common side effects of AI use include hot flashes, osteoporosis, arthralgias, fatigue, musculoskeletal pain, and hyperlipidemia [3-4]. We present the case of a 76-year-old female who presented with hepatic toxicity after initiating endocrine therapy with anastrozole for ER-positive lobular carcinoma in situ (LCIS). This rare case of anastrozole use leading to hepatic injury enhances understanding of the potential side effects and risks associated with AI use.

## Case presentation

A 76-year-old female with a past medical history of hypothyroidism and dyslipidemia presented to the clinic after routine screening mammogram showed abnormal microcalcifications of the left breast. Diagnostic mammogram demonstrated indeterminate microcalcifications of Breast-Imaging Reporting and Data System Category 4b in the left retroareolar region. Subsequent biopsy was significant for ER-positive pleomorphic LCIS with central necrosis. The patient underwent lumpectomy, which showed 3.5 millimeter pleomorphic LCIS that was ER 100% and progesterone receptor (PR)-negative with negative margins. Based on the characteristics of the tissue removed, radiation therapy was not indicated. The patient was offered endocrine therapy to reduce risk of recurrence or malignant transformation. She was agreeable to proceed with treatment and was started on anastrozole 1 milligram daily for a recommended duration of five years. 

Within days of starting anastrozole, the patient reported weakness, loss of appetite, and darkening of her urine. Within two weeks, she developed jaundice and was admitted to the hospital for evaluation. Her hepatic function panel demonstrated laboratory abnormalities, as shown in Table 1. The patient was diagnosed with acute liver injury and subsequently worked up to investigate an underlying cause. Imaging included ultrasound of the right upper quadrant, revealing cholelithiasis. Further evaluation with computed tomography of the abdomen and pelvis and magnetic resonance cholangiopancreatography demonstrated a normal gallbladder and biliary tree, normal pancreas, and no concerning liver lesions. Urinalysis and serology studies conducted are displayed in Table 2. Additional coagulation tests, complete blood count, antinuclear antibody test, and anti-smooth muscle antibody testing were within normal limits. Given the temporal association between anastrozole initiation and hepatic injury, along with an extensive workup ruling out infective or immune-mediated causes of injury, anastrozole was determined to be the source of the hepatic injury, and the medication was discontinued.

**Table 1:** Hepatic function panel results at hospital admission. g/dL, grams per deciliter; mg/dL, miligrams per deciliter; IU/L, international units per liter

Test	Result	Units	Reference interval
Albumin	3.9	g/dL	3.7-4.7
Total bilirubin	11.2	mg/dL	0.0-1.2
Direct bilirubin	9.62	mg/dL	0.00-0.40
Alkaline phosphatase (ALP)	1388	IU/L	44-121
Aspartate aminotransferase (AST)	384	IU/L	0-40
Alanine aminotransferase (ALT)	492	IU/L	0-32

**Table 2:** Laboratory study results obtained during hospitalization. mg/dL, miligrams per deciliter; HPF, high-power field; IU/L, International Units per liter; U/L, units per liter; g/dL, grams per deciliter

Test	Result	Units	Reference interval
Urinalysis with microscopy examination			
Color	Amber		Clear
Glucose	Negative	mg/dL	Negative
Bilirubin	1+		Negative
Ketones	20	mg/dL	Negative
Urobilinogen	2.0	mg/dL	<2.0
Leukocyte esterase	Trace		Negative
Leukocytes	10	/HPF	0-5
Enzymes			
Lipase	249	IU/L	73-393 IU/L
Creatine kinase (CK)	61	U/L	26-192
Gamma-glutamyl transferase (GGT)	1176 IU/L	IU/L	5-55 IU/L
Metabolic			
Ceruloplasmin	40 mg/dL	mg/dL	16-45 mg/dL
Alpha-1 antitrypsin (AAT)	197 mg/dL	mg/dL	90-200 mg/dL
Anion gap	6		7-16
Albumin, serum	2.4 g/dL	g/dL	3.4-5.0 g/dL
Phosphorus, inorganic	1.9	mg/dL	2.5-4.9 mg/dL
Infectious			
Cytomegalovirus (CMV) antibody (Ab) immunoglobulin M & G (IgM & IgG)	Negative		Negative
Hepatitis A Ab, IgM	Negative		Negative
Hepatitis B surface Ag (antigen)	Negative		Negative
Hepatitis B core Ab, total	Negative		Negative
Hepatitis C Ab	Negative		Negative
Human immunodeficiency virus (HIV) ½ Ag and Ab screen	Non-reactive		Non-reactive
Coagulation			
Protime + international normalized ratio (INR)	12.8	Seconds	12.0-15.0
Tumor markers			
Carbohydrate antigen (CA) 19-9	64.0		0.0-35.0

Two months after ceasing the medication, the patient’s symptoms resolved, and repeat laboratory values showed improvement, demonstrating mildly elevated total bilirubin 1.4 mg/dL (reference range 0.0-1.2 mg/dL), direct bilirubin 0.9 mg/dL (reference range 0.00-0.40 mg/dL), alkaline phosphatase (ALP) 229 IU/L (reference range 44-121 IU/L), and aspartate aminotransferase (AST) and alanine aminotransferase (ALT) within normal limits. Treatment alternatives with a selective estrogen receptor modulator or alternative AI were discussed with the patient. After explaining the benefits of therapy in reducing the recurrence of disease along with the potential risks of each agent, she decided to proceed with routine observation alone.

## Discussion

Drug-induced liver injury (DILI) is a spectrum of hepatotoxicity caused by medications or supplements that may present in a predictable dose-dependent pattern or idiosyncratically [5]. Unlike dose-dependent DILI, idiosyncratic DILI can be caused by immune-mediated or non-immune mediated reactions to the offending agent [6]. Consequently, diagnosis of DILI can be challenging as it may present similarly to other causes of inducible hepatotoxicity, notably autoimmune hepatitis (AIH) [7]. However, differentiation of these pathologies is crucial to proper management [7]. 

Third-generation AIs such as anastrozole, letrozole, and exemestane block the enzyme aromatase from converting androgens to estrogen, thus reducing the amount of estrogen produced by the body [2,8]. Since estrogen promotes the growth of hormone-sensitive breast cancers, estrogen-reducing agents demonstrate significant efficacy in reducing the recurrence of metastatic hormone-sensitive breast cancer [2]. Previous research has demonstrated that the use of AI for endocrine therapy in postmenopausal women is superior to tamoxifen by reducing the recurrence of disease at distant sites and increasing disease-free survival [9-10]. Estrogen produced by the ovaries will not be reduced by AI, limiting their use to endocrine therapy with confirmed postmenopausal status. However, tamoxifen demonstrates efficacy in reducing the risk of recurrence and death and may be used as an alternative to AI if contraindications for endocrine therapy exist [11]. Common side effects of AI include hot flashes, mood changes, decreased bone density, hyperlipidemia, arthralgias, fatigue, and musculoskeletal pain [3-4,8,12]. Although tamoxifen yields a different side effect profile than AI, the most important risks associated with tamoxifen use include venous thromboembolism and endometrial cancer [10-11]. 

Idiosyncratic DILI and AIH are very rare complications of anastrozole, the most frequently used AI for endocrine therapy, and have limited documented occurrences in medical literature [8,12-16]. Hepatotoxicity from DILI and AIH may present similarly, with symptoms, such as fatigue, weakness, dyspnea, jaundice, abdominal pain, altered mental status, and various laboratory abnormalities, most notably elevated liver enzymes. Autoimmune antibodies may provide diagnostic benefit as is characteristically associated with AIH, but may also be present in drug-induced autoimmune-like hepatitis subtype of DILI [8,12,14-15,17]. The timing of hepatotoxicity after the initiation of an offending agent is critical, as hepatic injury from AIH does not demonstrate an association to initiating therapy [8,12,17]. The histology of hepatic tissue demonstrates key distinctions and may be warranted if unable to determine a diagnosis [17]. Both pathologies may present with increased infiltrate of lymphocytes and plasma cells; however, advanced fibrosis or cirrhosis is characteristic to AIH and largely not seen in DILI [8,14,17]. 

Further support for a diagnosis of DILI can be made by utilizing the Roussel Uclaf Causality Assessment Method (RUCAM), a validated system used to evaluate the likelihood of a specific medication inducing liver injury [18]. This model scores various factors including pattern of liver injury, time to the onset of symptoms, illness course, risk factors, concomitant medications, non-drug causes of injury, and recurrent injury with repeat medication exposure [18]. The RUCAM model categorizes the likelihood of medication induced liver injury as “excluded," “unlikely," “possible," “probable," or “highly probable” based on predefined scoring criteria [18]. In this patient’s case, a RUCAM score of 7 was calculated, indicating a probable association between anastrozole and hepatic injury.

The management of DILI involves the removal of the offending agent with possible glucocorticoid adjunct depending on the severity of injury [17]. Following discontinuation, hepatic injury is largely reversible with the resolution of symptoms and return of liver function enzymes to normal limits in one to two months [12,15,17]. Treatment for AIH involves long-term therapy with glucocorticoids and azathioprine [17]. Due to the rarity of hepatotoxicity from AI, subsequent management for endocrine therapy is poorly understood. One reported case changed endocrine therapy to tamoxifen for the remainder of the therapy duration and demonstrated no disease recurrence [12]. Considerations for altering endocrine therapy regimen should include the risk of DILI with other AIs, side effect profile, and risk for recurrence and metastasis. After discussing the risks and benefits of each therapy, our patient accepted the risk of recurrence and declined restarting endocrine therapy, electing to proceed with routine observation alone.

## Conclusions

This case highlights the rare occurrence of hepatotoxicity induced by anastrozole, broadening the current understanding of the side effect profiles and risks of AI. In this patient, discontinuation of anastrozole resulted in significant improvement of liver function tests, and the patient decided after a discussion of the risks and benefits of endocrine therapy to proceed with regular observation. This case reinforces the importance for accurate understanding, recognition, and diagnosis of hepatic injury for proper medical management, especially due to limited documented cases of DILI with AI.

## References

[1] Giaquinto AN, Sung H, Newman LA (2024). Breast cancer statistics 2024. CA Cancer J Clin.

[2] Bhatia N, Thareja S (2024). Aromatase inhibitors for the treatment of breast cancer: an overview (2019-2023). Bioorg Chem.

[3] Lønning PE (2011). The potency and clinical efficacy of aromatase inhibitors across the breast cancer continuum. Ann Oncol.

[4] (2025). Label: ARIMIDEX- anastrozole tablet. https://dailymed.nlm.nih.gov/dailymed/drugInfo.cfm?setid=4fee8d4c-bb21-4773-b622-87f9a4ff333a.

[5] Iorga A, Dara L, Kaplowitz N (2017). Drug-induced liver injury: cascade of events leading to cell death, apoptosis or necrosis. Int J Mol Sci.

[6] Jee A, Sernoskie SC, Uetrecht J (2021). Idiosyncratic drug-induced liver injury: mechanistic and clinical challenges. Int J Mol Sci.

[7] Andrade RJ, Aithal GP, de Boer YS (2023). Nomenclature, diagnosis and management of drug-induced autoimmune-like hepatitis (DI-ALH): an expert opinion meeting report. J Hepatol.

[8] Klapko O, Ghoulam E, Jakate S, Eswaran S, Usha L (2017). Anastrozole-induced autoimmune hepatitis: a rare complication of breast cancer therapy. Anticancer Res.

[9] Iwase H (2008). Current topics and perspectives on the use of aromatase inhibitors in the treatment of breast cancer. Breast Cancer.

[10] Janni W, Hepp P (2010). Adjuvant aromatase inhibitor therapy: outcomes and safety. Cancer Treat Rev.

[11] Lin NU, Winer EP (2008). Advances in adjuvant endocrine therapy for postmenopausal women. J Clin Oncol.

[12] Inno A, Basso M, Vecchio FM (2011). Anastrozole-related acute hepatitis with autoimmune features: a case report. BMC Gastroenterol.

[13] Lischka W, Kriegshäuser G (2024). Drug-induced liver injury as assessed by the updated Roussel Uclaf Causality Assessment Method following mild COVID-19 in a patient under anastrozole therapy-a case report. Cancer Rep (Hoboken).

[14] Islam MS, Wright G, Tanner P, Lucas R (2014). A case of anastrazole-related drug-induced autoimmune hepatitis. Clin J Gastroenterol.

[15] de la Cruz L, Romero-Vazquez J, Jiménez-Sáenz M, Padron JR, Herrerias-Gutierrez JM (2007). Severe acute hepatitis in a patient treated with anastrozole. Lancet.

[16] Zapata E, Zubiaurre L, Bujanda L, Piérola A (2006). Anastrozole-induced hepatotoxicity. Eur J Gastroenterol Hepatol.

[17] Mack CL, Adams D, Assis DN (2020). Diagnosis and management of autoimmune hepatitis in adults and children: 2019 practice guidance and guidelines from the American Association for the Study of Liver Diseases. Hepatology.

[18] Danan G, Teschke R (2015). RUCAM in drug and herb induced liver injury: the update. Int J Mol Sci.

